# Temperature and nitrogen supply interact to determine protein distribution gradients in the wheat grain endosperm

**DOI:** 10.1093/jxb/ery127

**Published:** 2018-04-05

**Authors:** George P Savill, Adam Michalski, Stephen J Powers, Yongfang Wan, Paola Tosi, Peter Buchner, Malcolm J Hawkesford

**Affiliations:** 1 Plant Sciences Department, Rothamsted Research, West Common, Harpenden, UK; 2 Institute of Geodesy and Geoinformatics, Wrocław University of Environmental and Life Sciences, ul. Grunwaldzka, Wrocław, Poland; 3 Computational and Analytical Sciences Department, Rothamsted Research, West Common, Harpenden, UK; 4 School of Agriculture, Policy, and Development, University of Reading, Earley, Reading, UK

**Keywords:** Breadmaking quality, gluten protein, grain protein distribution, protein body size distribution, protein distribution gradient, *Triticum aestivum*, wheat endosperm, wheat grain protein

## Abstract

Gradients exist in the distribution of storage proteins in the wheat (*Triticum aestivum*) endosperm and determine the milling properties and protein recovery rate of the grain. A novel image analysis technique was developed to quantify both the gradients in protein concentration, and the size distribution of protein bodies within the endosperm of wheat plants grown under two different (20 or 28 °C) post-anthesis temperatures, and supplied with a nutrient solution with either high or low nitrogen content. Under all treatment combinations, protein concentration was greater in the endosperm cells closest to the aleurone layer and decreased towards the centre of the two lobes of the grain, i.e. a negative gradient. This was accompanied by a decrease in size of protein bodies from the outer to the inner endosperm layers in all but one of the treatments. Elevated post-anthesis temperature had the effect of increasing the magnitude of the negative gradients in both protein concentration and protein body size, whilst limiting nitrogen supply decreased the gradients.

## Teaser

Nitrogen supply and post-anthesis temperature affect the protein distribution gradient in the wheat grain endosperm, as measured using a novel image analysis technique for light microscopy sections.

## Introduction

The starchy endosperm is the central storage tissue of the wheat grain and is the material from which white flour is produced. Gradients exist in the distribution of gluten storage proteins within the starchy endosperm of developing and mature grain: these gradients are both qualitative and quantitative in nature (Tosi *et al.*, 2011; He *et al.*, 2013), and influence both the milling properties and milling yield of the grain. The milling of wheat grain is achieved through a sequence of milling and sieving stages, which results in the production of numerous mills streams. These individual mill streams are enriched with different parts of the wheat grain, and so gradients in endosperm protein concentration and composition will result in the production of mill streams with specific protein content, composition, and baking characteristics (Yahata *et al.*, 2006; Wang *et al.*, 2007; Wan *et al.*, 2014; Zhou *et al.*, 2018). Different mill streams are then blended by millers to produce flour with the desired qualities, and so understanding the factors affecting the composition of mill streams is of great importance. Furthermore, during milling a proportion of the endosperm tissue remains adhered to the bran layers, and since protein is concentrated in the outer layers of the endosperm (Kent, 1966; Ugalde and Jenner, 1990*a*; Tosi *et al.*, 2011; He *et al.*, 2013), a proportionally greater amount of protein relative to starch is lost during the production of white flour. Hence, any increase in the gradient of protein concentration may result in an even greater amount of protein being lost during milling.

Whilst the presence of a gradient in protein concentration within the wheat grain has been known for some time (Cobb, 1905), the mechanisms behind this gradient remain unexplained. Recently Moore *et al.* (2016) used ^15^N labelling with NanoSIMS analysis to demonstrate that glutamine, the most abundant form of nitrogen within the wheat plant (Fisher and Macnicol, 1986), is transported radially from the endosperm cavity, across the starchy endosperm, before becoming concentrated in the subaleurone cells. This study, which focussed on limited microscale transects, demonstrated that the subaleurone cells had larger protein bodies, and it was speculated that they acted as a strong sink driving amino acid transport across the endosperm.

The future of wheat production for bread-making is likely to face many challenges, such as limits on the use of nitrogen fertiliser and changing climate, which, in the UK, will include increases in ambient temperatures experienced during grain filling. The production of nitrogen fertiliser is intensive in its energy requirement, and at present relies heavily on the use of fossil fuels (Dawson and Hilton, 2011). Due to this, both financial and policy constraints are likely to limit the amount of nitrogen fertiliser that can be applied to future bread-making wheat crops. Additionally, it is predicted that there will be a global increase in temperature (Alexander *et al.*, 2006) and an increase in temperature variability, with more frequent extreme weather events such as heatwaves and droughts (Hennessy *et al.*, 2008); these changes to our climate are likely to be to the detriment of agriculture and food security (Wheeler and von Braun, 2013)

Elevated temperature affects the development of wheat, drastically shortening the grain-filling period, and resulting in altered grain expression and related protein accumulation (Altenbach, 2012). Nitrogen nutrition has been shown to affect the deposition of protein within the grain, with higher levels of nitrogen supply resulting in an increase in ω-gliadin accumulation in the outer layers of the starchy endosperm (Wan *et al.*, 2014). The effect of temperature on the gradients of gluten storage proteins within the wheat endosperm has not been previously described.

Previous analysis of protein gradients in wheat grain has been a low-throughput process, reliant on micro-dissection (Cobb, 1905; Ugalde and Jenner, 1990*a*, 1990*b*), sub-sampling of microscopy images (Tosi *et al.*, 2011), or pearl milling (He *et al.*, 2013). Here, a high-throughput image analysis technique for quantifying features in light microscopy sections is presented, which analyses both the gradient in protein concentration and the size distribution of protein bodies within the endosperm of wheat grain during grain-filling. Wheat grain grown under controlled-environment conditions is used to describe the effect of elevated post-anthesis temperature and reduced nitrogen input on storage protein accumulation within the wheat endosperm in the context of UK climate and agronomical practice.

## Materials and methods

### Plant material and growth conditions

A spring wheat cultivar (*Triticum aestivum* L., ‘Cadenza’) was grown under controlled-climate conditions at Rothamsted Research, Harpenden, UK. A complete randomised block design was used to grow 480 plants in 96 pots split equally between three experimental blocks, with a treatment structure of two levels of nitrogen fertiliser crossed with two post-anthesis temperature regimes.

Plants were grown to anthesis in a single Weiss Gallenkamp controlled-environment room with a floor area of 16 m^2^ and a height of 3 m. Day/night temperatures were set at 20/15 °C and humidity at 65/75%. The photoperiod was maintained at 16 h per day, and the light intensity was 500 µmol m^–2^ s^–1^ from 400 W HQI metal halide lamps with a supplementary 10% mix of tungsten lighting. The change between day and night temperatures was programmed to take 2 h, to approximate field conditions. At anthesis, which was determined when three out of five plants in a pot showed externally visible anthers, half of the plants were moved to a second, identical controlled-environment room and grown at day/night temperatures of 28/15 °C. This temperature regimen was chosen since it represents the minimum threshold temperatures for heatwaves in the UK (Public Health England, 2014). The remaining plants were kept in the original room at the control day/night temperature of 20/15 °C. Humidity and photoperiod remained unchanged between treatments, and the layout of pots within the room was maintained throughout.

Plants were grown in 25-cm pots with the nutrient-poor ‘Rothamsted nematode mix’ (80% sterilised loam, 15% sand, and 5% 5-mm grit), supplemented with either a high- or low-nitrogen liquid nutrient solution. Nutrient solution was applied from 10 d after sowing, at a rate of 500 ml once a week until ear emergence, and 500 ml twice a week from ear emergence to anthesis. Nine applications (4.5 l in total) of nutrient solution were made per pot of five plants, supplying 504 mg and 50.4 mg of nitrogen to the high- and low-nitrogen treatments respectively. The composition of the nutrient solutions was 4 mM Ca(NO_3_)_2_.4H_2_O (high-nitrogen treatment only), 0.4 mM Ca(NO_3_)_2_.4H_2_O (low-nitrogen treatment only), 3.6 mM CaCl_2_ (low-nitrogen treatment only), 0.25 mM KH_2_PO_4_, 0.5 mM KOH, 0.75 mM MgSO_4_.7H_2_O, 0.03 mM CaCl_2_, 0.1 mM FeNaEDTA, 30 µM H_3_BO_3_, 10 µM MnSO_4_.4H_2_O, 1 µM ZnCl_2_.7H_2_O, 3 µM CuSO_4_.5H_2_O, and 0.5 µM Na_2_MoO_2_.2H_2_O. Pots were placed on saucers to prevent loss of nutrient solution, and plants were watered with deionised water as required to prevent the soil from drying out.

### Sampling protocol

Grain was sampled for light-microscopy analysis from the first floret in spikelets from the centre of the ear, to ensure grain of comparable developmental status were selected for analysis. Samples were taken from the first ear to reach anthesis on each plant. One developing grain per ear and treatment combination per block (three replicates) was harvested at 14 and 21 d post-anthesis for the low-temperature (20 °C) treatment, and at 10 and 15 d post-anthesis for the high-temperature (28 °C) treatment. Sampling timepoints were adjusted to account for the difference in accumulated thermal time experienced by the plants in each treatment. Accumulated thermal time was calculated using a base temperature of 4.1 °C (Fischer, 1985), and rounded to the nearest day to determine sampling timepoints. Sixteen grains were also sampled for total nitrogen content analysis at the same timepoints used for microscopy analysis.

### Morphological and nitrogen content measurements

At maturity, all five plants within a pot were harvested, hand-threshed, and dried at 80 °C to 4–5% moisture content. Grain from each pot was combined and used to measure thousand grain weight, grain yield, and final nitrogen content. A total grain count was calculated from the grain yield and thousand grain weight.

Nitrogen content of the wholemeal flour was determined by the Dumas method using a LECO CN628 Combustion Analyser (LECO Corporation, St Joseph, Michigan, USA), and is expressed as percent dry matter.

### Microscopy

Developing grain sampled at two timepoints during grain development were fixed in 4% paraformaldehyde, 2.5% glutaraldehyde fixative, dehydrated in a graded ethanol series and embedded in medium-grade LR White Resin as described by Tosi *et al.* (2011). Sections of 1 µm thickness were cut and stained for protein with 1% Naphthol Blue Black in 7% (v/v) acetic acid for 30 s. Images were captured at 20× magnification on a Zeiss Axiophot light microscope (Zeiss, Oberkochen, Germany), and stitched into high-resolution composite images using MetaMorph Microscopy Automation and Image Analysis Software (Molecular Devices, Sunnyvale, CA, USA) and Adobe Photoshop (Adobe Systems Inc., San Jose, CA, USA). Composite images of the entire endosperm were then analysed.

### Image analysis

**Fig. 1.**
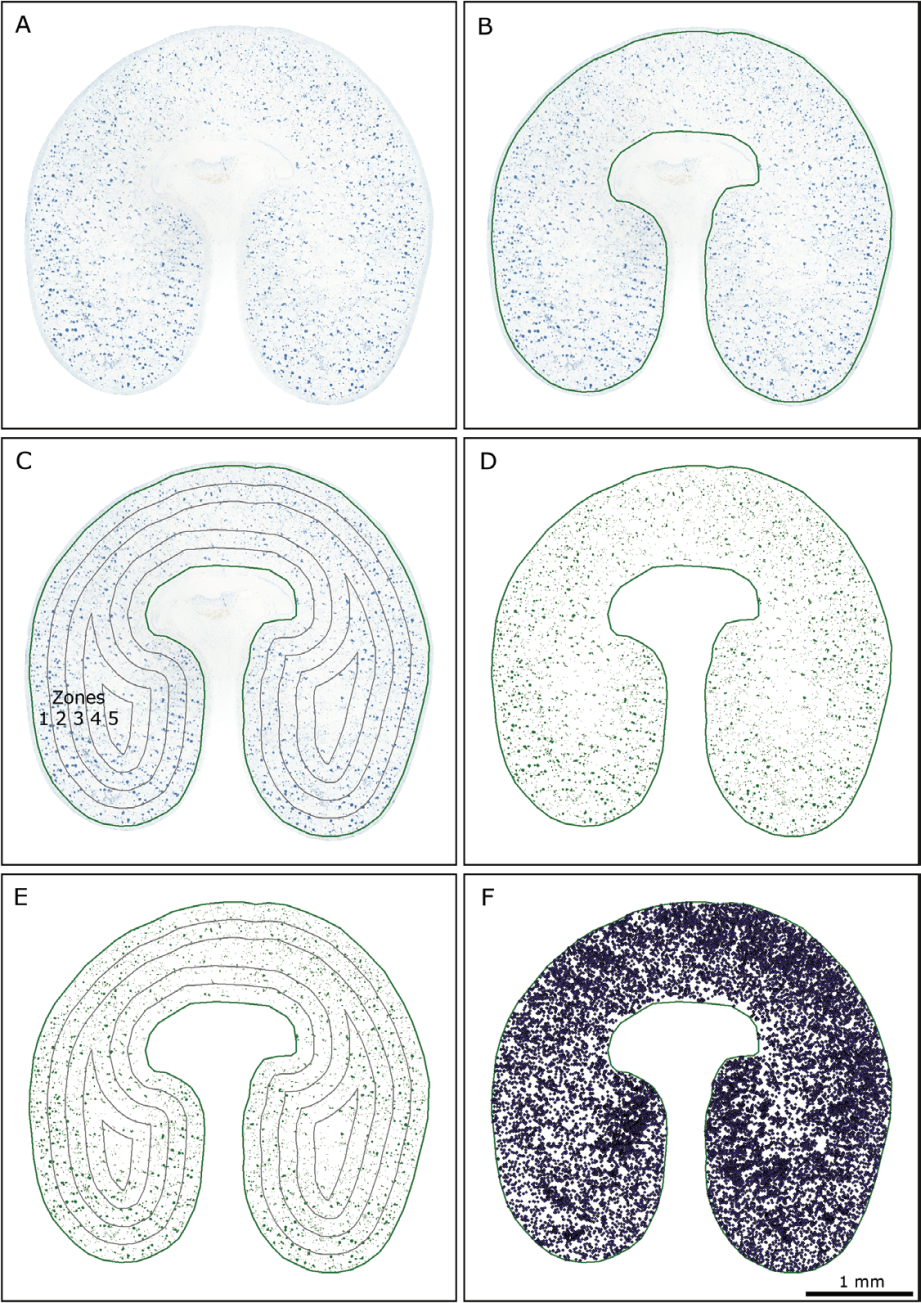
Overview of the grain protein gradient analysis procedure. (A) A composite light-microscopy image of a 1-µm thick wheat grain section selectively stained for protein with 1% Naphthol Blue Black in 7% acetic acid (v/v) is loaded. (B) An outline is manually drawn around the endosperm, just inside the aleurone layer. (C) Five concentric bands are automatically drawn inwards from the outline of the endosperm, creating zones representing tissue from outer to inner endosperm (labelled 1–5). (D) Pixels representing protein are extracted using image classification, and the background is removed. (E) The zones from stage (C) are overlaid on the extracted protein from stage (D) and protein concentration values are calculated on an area basis for each zone within the endosperm. (F) For protein body size distribution analysis, individual protein bodies are detected, and their area and distance from the aleurone layer are measured. The example shown is a section from a grain under the high-nitrogen, 20 /15 °C temperature treatment, sampled at 21 d post-anthesis.

Light-microscopy images of developing wheat grain stained for protein were analysed using a custom toolbox in ArcMap™, part of the ArcGIS™ 10.4 software package (ESRI®, Redlands, CA, USA) (available as an ArcGIS™ python toolbox, doi: 10.5281/zenodo.1066914). Images were loaded (Fig. 1A), and an outline was manually drawn around the endosperm of the grain, just within the aleurone layer (Fig. 1B). Two methods of image analysis were then run simultaneously: protein concentration distribution analysis, and protein body size distribution analysis. Protein concentration distribution analysis describes the overall gradients in protein concentration, whilst protein body size distribution analysis measures the area of each individual protein body, and records the distance of that protein body from the outline drawn around the endosperm.

Supervised maximum likelihood classification was used to detect protein within the light-microscopy images on a pixel-by-pixel basis (Fig. 1D). For this classification, multiple training samples were taken from each RGB image to give examples of both stained protein, and non-protein areas within the endosperm. These training samples were taken from 10 areas of protein across different parts of the grain to account for variation in stain intensity across the section, and from sufficient non-protein areas of the grain to account for all combinations of tone and intensity that did not represent protein. To account for variation in the defined training samples used for the image classification, each image was analysed three times using different training samples each time. Furthermore, every analysis instance was checked to verify the accuracy of the image classification in discriminating protein from background.

To describe the gradient in protein within the endosperm, protein concentration distribution analysis was used to measure and compare protein concentration in five zones within the endosperm. Five concentric bands were automatically rendered inwards from the endosperm outline to create five zones of equal width covering the range of outer to inner endosperm (Fig. 1C). These zones were then overlaid on the extracted protein body area data as determined by maximum likelihood classification (Fig. 1D), and protein within each zone was measured. The result is a measurement of protein concentration on a by-area basis within each of the five zones in the endosperm (Fig. 1E).

The use of five zones was determined to be optimal through experimentation with different numbers of zones (from three to seven) to find the number that was geometrically optimal to describe the gradient in protein concentration via best-fit regression modelling of total protein body area per zone versus zone number (data not shown).

For protein body size distribution analysis, the area of each protein body was measured, the midpoint detected (Fig. 1F), and the shortest Euclidean distance between this point and the outline of the endosperm calculated.

### Data processing

Post-analysis, a conversion was applied to the data collected from the protein concentration gradient analysis: nitrogen content measurements made on grain sampled from the same plant and at the same timepoint were used to calculate a conversion factor between actual grain protein content and protein content as determined through microscopy-image analysis. A unique conversion factor was calculated for each combination of treatment and experimental replicate. This conversion factor was applied to all collected data to account for any differences in protein detection efficiency between treatments, since any changes in grain water content or protein density within a protein body would result in inaccuracies in the measurement of protein concentration on a by-area basis. The equation used for the conversion was as follows:

$$\text{Conversion}\;\text{factor}=\frac{\text{Protein}\;\text{concentration}\;\text{from}\;\text{nitrogen}\;\text{content}\;\text{measurements}}{100\;\times \left(\frac{\text{Total}\;\text{protein}\;\text{area}\;\text{from}\;\text{microscopy}\;\text{image}\;\text{analysis}}{\text{Total}\;\text{grain}\;\text{area}\;\text{from}\;\text{microscopy}\;\text{image}\;\text{analysis}}\right)}$$

To account for differences in the size of grain from different treatments, prior to analysis the zone attributed to each protein concentration measurement was converted from a factor to a variable by calculating the distance between the outline of the endosperm and the midpoint between the boundaries of each zone. This value was then converted from pixels to micrometres to give a meaningful mean physical distance for each of the five protein concentration measurements made on each image.

### Statistical analysis

The GenStat® statistical software (2015, Eighteenth Edition, VSN International Ltd, Hemel Hempstead, UK) was used to analyse the effect of post-anthesis temperature and nitrogen fertiliser level on the total grain count, nitrogen yield, thousand grain weight, and total grain yield by ANOVA. After inspection of *F*-tests for the main effects and interactions between the treatment factors, the least-significant difference at the 5% (*P*=0.05) level calculated from the standard error of the difference between means on the residual degrees of freedom from the ANOVA was used to make comparisons of relevant means.

GenStat® was used to fit a linear mixed model to both the distribution of protein concentration over the calculated zones, and to the protein body size distribution data using the method of restricted maximum likelihood. Data from each image analysis technique were analysed separately.

In the analysis of the protein concentration distribution data, the calculated mean distance from the aleurone cell layer within the wheat grain was used rather than the zone factor. The fixed part of the linear mixed model was Temperature*Nitrogen*Timepoint*Distance, where *Temperature*, *Nitrogen*, and *Timepoint* are factors, *Distance* is a variable calculated from zone, and asterisks represent the main effects and interactions between the model terms. In the analysis of the protein body size distribution data, the protein body area measurements were log-transformed to account for some heterogeneity of variance over the treatment combinations. The fixed part of the linear mixed model was Temperature*Nitrogen*Timepoint*ProteinDistance, where *Temperature*, *Nitrogen*, and *Timepoint* are factors, and *ProteinDistance* is a variable. The random part of each model was (*Room.Block*)/*Pot*/*ImageRep*/*Zone*/*AnalysisRep* and (*Room.Block*)/*Pot*/*ImageRep* /*AnalysisRep* for protein concentration and body size, respectively, where *Room.Block* is the interaction between growth rooms and experimental blocks to account for the two rooms being used, *Pot* is the factor for pots within room by block combinations, *ImageRep* is the technical replication of microscopy sections, *Zone* is the concentric zone within the endosperm of each image from which a measurement was taken, *AnalysisRep* is the technical replication of each image analysis, and slash (/) indicates nesting of these factors. Non-linearity with respect to distance of measurement from the aleurone was tested by adding a distance-squared term to the fixed part of the model, and no evidence of non-linearity was found (*P*<0.05, *F*-tests). Inspection of residual plots revealed that log-transformation of the protein body area data was adequate to stabilise their variance, and that no transformation was required for any of the other analyses.

## Results

### Measurements taken at harvest

**Fig. 2.**
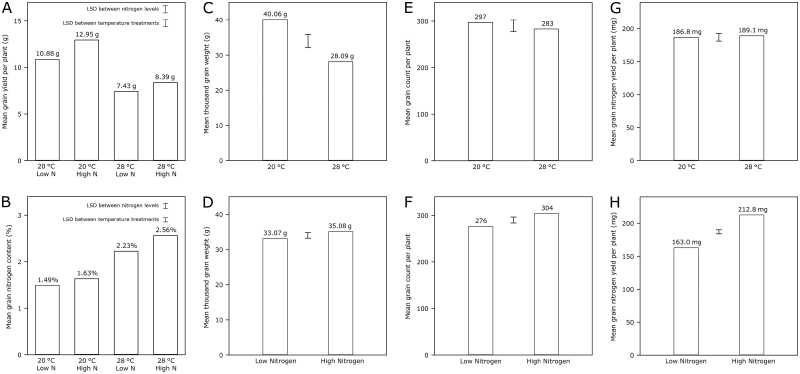
Mature grain characteristics. (A) Mean grain yield (*n*=3) per plant for all combinations of temperature and nitrogen treatments. (B) Mean grain nitrogen content (*n*=3) for all combinations of temperature and nitrogen treatments. (C) Mean thousand grain weights (*n*=6) for the two temperature treatments, and (D) for the two nitrogen treatments (*n*=6). (E) Calculated mean total grain counts (*n*=6) per plant for the two temperature treatments, and (F) for the two nitrogen treatments (*n*=6). (G) Calculated mean grain nitrogen yield (*n*=6) per plant for the two temperature treatments, and (H) for the two nitrogen treatments (*n*=6). Bars for least-significant differences (*P*=0.05) are shown.

Grain yield was reduced by both increased post-anthesis temperature and reduced nitrogen supply, with a significant interaction between these two factors (*P*=0.008, *F*-test): reducing the nitrogen application rate had a greater effect on yield in plants grown under 20 /15 °C post-anthesis temperature conditions than in plants grown under 28 /15 °C post-anthesis temperature (Fig. 2A). Yield was higher under the control temperature treatment, regardless of nitrogen input level.

Thousand grain weight was reduced by an average of 11.97 g, from 40.06 g to 28.09 g, under high post-anthesis temperature (*P*<0.001, *F*-test) (Fig. 2C) and by 2.01 g, from 35.08 g to 33.07 g, under the low-nitrogen treatment (*P*=0.019, *F*-test) (Fig. 2D). No significant interaction between temperature and nitrogen treatment was found (*P*=0.170, *F*-test).

Mean grain count per plant was calculated, and was found to be reduced by approximately 10%, from 304 to 276 grains per plant, under the low-nitrogen treatment (*P*=0.002, *F*-test) (Fig. 2F); elevated post-anthesis temperature did not affect grain count (*P*=0.198, *F*-test) (Fig. 2E), and there was no interaction between nitrogen and temperature treatments (*P*=0.723, *F*-test).

Nitrogen concentration at maturity was higher in grain grown under elevated post-anthesis temperature, and in grain grown under higher levels of applied nitrogen, with a significant interaction between these two factors (*P*=0.032, *F*-test): increasing nitrogen application rate increased grain nitrogen concentration to a greater extent in plants grown under high post-anthesis temperature (from 2.23% to 2.56%) than in plants grown under control conditions (from 1.49% to 1.63%) (Fig. 2B). Grain nitrogen yield per plant was calculated from total grain yield and nitrogen concentration measurements, and was reduced by the low-nitrogen treatment (*P*<0.001, *F*-test) (Fig. 2H), but was not significantly different between post-anthesis temperature treatments (*P*=0.626, *F*-test) (Fig. 2G), and there was no significant interaction between nitrogen and temperature treatments (*P*=0.649, *F*-test).

Time to maturity was greatly reduced by an increased temperature post-anthesis, with an average time from anthesis to harvest at maturity of 46 d for the control temperature treatment, and 33 d for the elevated temperature treatment.

### Grain protein concentration gradients

**Fig. 3.**
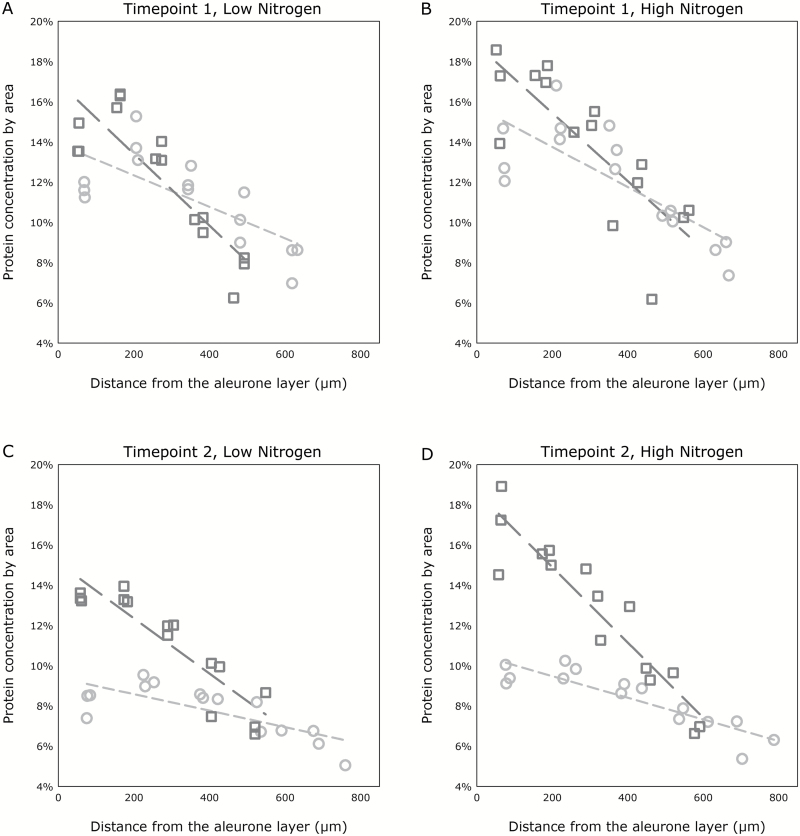
Results of protein concentration gradient analysis. Grain sampled at timepoint 1 with low-nitrogen treatment (A) and high-nitrogen treatment (B), and at timepoint 2 with low-nitrogen treatment (C) and high-nitrogen treatment (D). Dark grey squares and long-dashed lines represent the 28 °C treatment, and light grey circles and short-dashed lines represent the control 20 °C treatment. The graphs in (A–D) show results of the same model, with portions of the results being separated for clarity. Trendlines represent the result of the linear mixed modelling analysis (using restricted maximum likelihood), which was applied to the entire dataset, whilst data points represent mean protein concentration values within each zone from each of the three experimental replicates analysed. Underlying biological variation for entire dataset, *s*^2^=0.178.

A significant four-way interaction was found between post-anthesis temperature, nitrogen treatment, sampling timepoint, and mean distance of protein concentration from the aleurone layer (*P*=0.049, *F*-test). Under all treatments, protein concentration was greatest nearest to the aleurone layer, and lowest farthest from the aleurone layer. This negative gradient in protein concentration was greater, with more protein located closer to the aleurone layer, both in grain grown under high temperatures, and in grain grown under high nitrogen. The effect of nitrogen supply on the gradient was greater in grain grown under higher temperatures. Furthermore, the negative gradient in protein concentration was greater, and the effect of both elevated post-anthesis temperature and nitrogen supply more pronounced at the later sampling timepoint. The protein concentration gradient results for low- and high-nitrogen treatments at the first sampling timepoint are shown in Fig. 3A and 3B, respectively, and Fig. 3C and 3D show the same for the second, later sampling timepoint.

### Protein body size distribution

A significant four-way interaction was found between post-anthesis temperature treatment, nitrogen treatment, sampling timepoint, and distance of protein bodies from the aleurone layer (*P*<0.001, *F*-test). Under all treatment combinations, a gradient in protein body size was observed, with mean size increasing towards the aleurone layer in every treatment combination except for the control temperature, low-nitrogen, later sampling timepoint treatment combination, where protein bodies showed a marginal decrease in size towards the aleurone layer. Protein bodies were larger at the later sampling timepoint for all treatments, and were larger in grain grown under control temperature at the earlier timepoint, but larger in grain grown at high temperature at the later timepoint. At the later sampling timepoint the effects of both post-anthesis temperature and nitrogen supply on protein body size distribution were more pronounced. Across both temperature treatments, grain provided with a higher supply of nitrogen showed greater gradients in the size distribution of protein bodies, with larger protein bodies closer to the aleurone layer, and smaller protein bodies towards the centre of the endosperm.

**Fig. 4.**
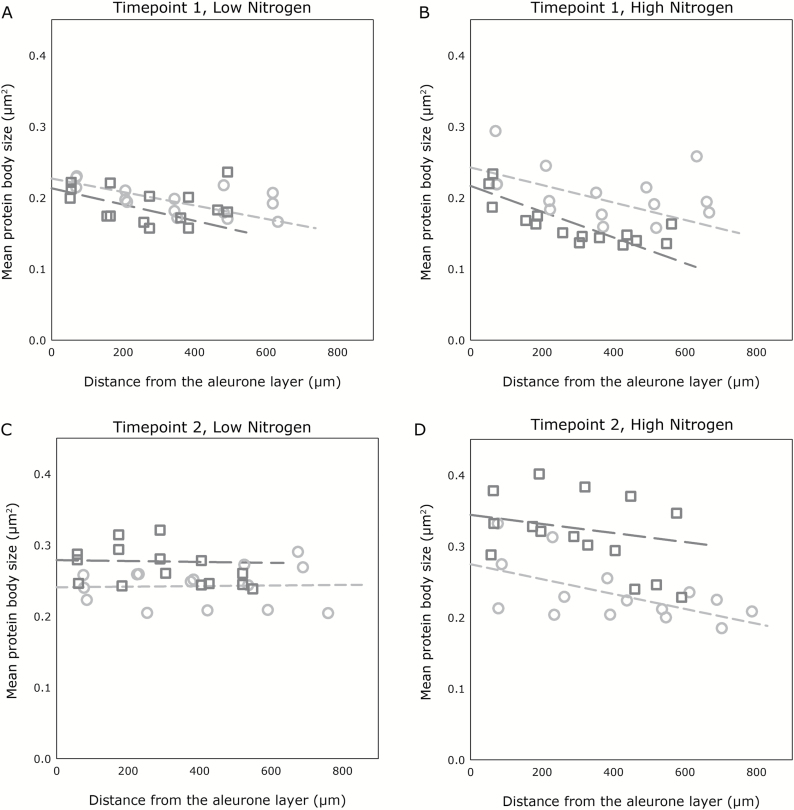
Results of protein body size distribution gradient analysis. Grain sampled at timepoint 1 with low-nitrogen treatment (A) and high-nitrogen treatment (B), and at timepoint 2 with low-nitrogen treatment (C) and high-nitrogen treatment (D). Dark grey squares and long-dashed lines represent the 28 °C treatment, and light grey circles and short-dashed lines represent the control 20 °C treatment. The graphs in (A–D) show results of the same model, with portions of the results being separated for clarity. Trendlines represent the result of the linear mixed modelling analysis (using restricted maximum likelihood), which was applied to the entire dataset. This analysis found that separate linear trends was the best statistical representation of the temperature by nitrogen by timepoint interactions. Data points represent mean protein body size from each of the three experimental replicates within each zone. The analysis was completed on log-transformed data, and back-transformed data is presented for clarity. Underlying biological variation for entire dataset, *s*^2^=2.345.

**Fig. 5.**
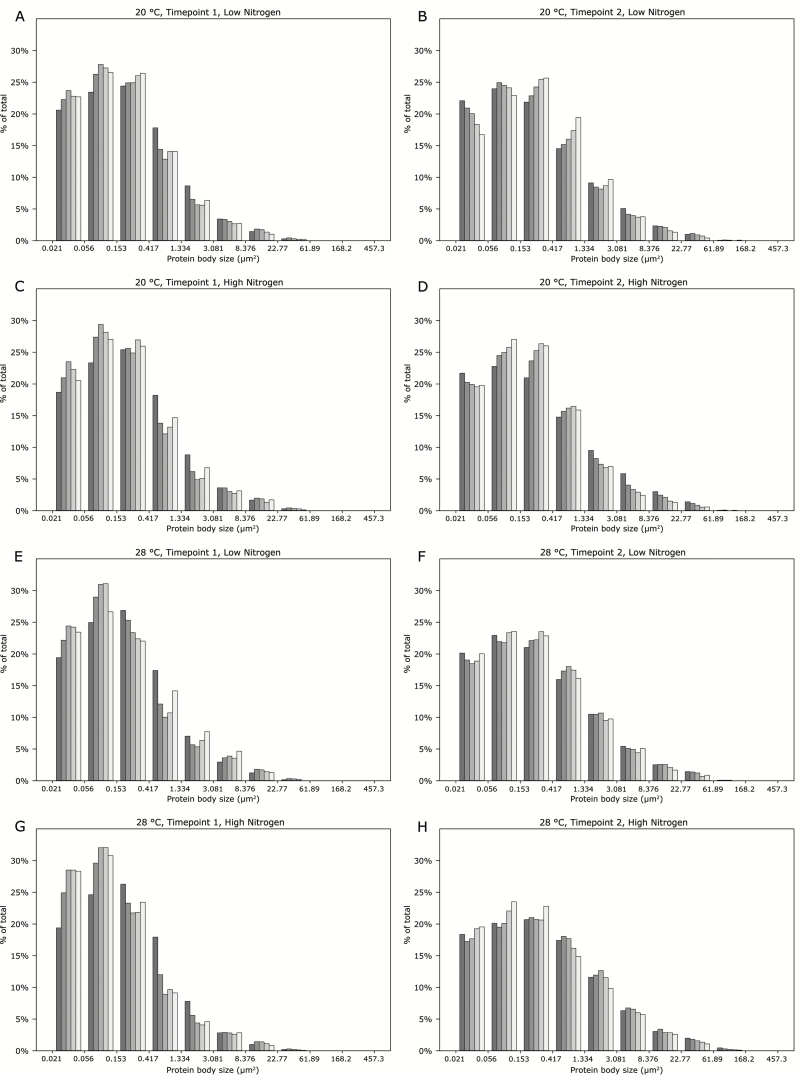
Histograms showing the frequency of protein body sizes across each of the five endosperm zones. Grain was (A) grown at 20 °C with low-nitrogen treatment and sampled at timepoint 1, and (B) sampled at timepoint 2; (C) grown at 20 °C with high-nitrogen treatment and sampled at timepoint 1, and (D) sampled at timepoint 2; (E) grown at 28 °C with low-nitrogen treatment and sampled at timepoint 1, and (F) sampled at timepoint 2; (G) grown at 28 °C with high-nitrogen and sampled at timepoint 1, and (H) sampled at timepoint 2. Data for zones 1–5 (see Fig. 1) are represented within each graph by shades of grey from dark (zone 1) to light (zone 5), and are the same zones that were used to plot the means in Fig. 4.

The results of the protein body size distribution analysis are presented in Fig. 4A and 4B for the low- and high-nitrogen treatments, respectively at the first sampling timepoint, and in Fig. 4C and 4D for the later sampling timepoint. The plotted means for grain sampled at the earlier timepoint (Fig.4A, B) show an initial decrease, followed by some evidence of an increase in mean protein body size as the distance from the aleurone layer increases beyond ~500 µm. This effect is only observed at the early sampling timepoint, with the plotted means from the later timepoint showing a more linear relationship with distance from the aleurone layer. These results are explored further in the histograms of protein body size distribution across the five endosperm zones shown in Fig. 5, which show how size is affected by each combination of temperature and nitrogen treatment at both the early and late sampling timepoint.

## Discussion

### Grain morphology

Elevated post-anthesis temperature produced the greatest change in grain morphology, with grain subjected to this treatment showing a greatly reduced total yield and thousand grain weight (Fig. 2A, C). These results indicate that the size of the mature grain was greatly reduced by increased temperatures during grain-filling. Limiting nitrogen supply prior to anthesis also reduced the grain size (Fig. 2D), albeit to a lesser degree than elevated temperature, and the effect of nitrogen input on grain yield was less apparent in plants grown under elevated post-anthesis temperatures (Fig. 2A).

As the elevated temperature treatment was only applied post-anthesis, the grain set was unaffected by the high temperature treatment. In contrast, nitrogen input did influence grain setting, with fewer grain being produced when nitrogen supply was limited. This shows how the effect of decreased nitrogen has a twofold impact on grain yield: reducing both grain number and grain size. However, the yield reduction caused by limiting nitrogen input can be primarily accounted for by the reduction in grain set rather than a decrease in grain size.

### Grain nitrogen

Elevated post-anthesis temperature resulted in an increase in grain protein concentration, whilst limiting the supply of nitrogen during vegetative development had the result of decreasing the concentration of protein in the mature grain. The effect of decreased nitrogen supply on the grain protein concentration was greater in plants subjected to high post-anthesis temperatures than those grown under control temperatures, suggesting an increased sensitivity to nitrogen input at higher temperatures, and confirming previous reports (Bhullar and Jenner, 1985; Gooding, 2010). However, elevated post-anthesis temperature treatment did not have an impact on the yield of protein in the grain: the same physical amount of protein accumulated in the grain regardless of temperature. Hence, elevated temperature during grain-filling is primarily impacting on the accumulation of starch, resulting in a greater concentration of protein in the grain, as previously reported by Altenbach *et al.* (2003), Dupont *et al.* (2006), and Koga *et al.* (2015).

### Microscopy analysis

The analysis of developing grain taken from the centre of the ear using light microscopy showed that both post-anthesis temperature and nitrogen supply interacted to affect the accumulation of protein within the endosperm during grain-filling, and that this was accompanied by changes in the size distribution of protein bodies.

Higher post-anthesis temperature resulted in grain with higher overall protein concentration and steeper protein gradients across the grain, i.e. protein was disproportionally accumulated in the outer layers of the endosperm. These outer endosperm cells may adhere to the aleurone layer during milling, and hence if heatwave conditions were experienced during grain-filing it is possible that high grain protein content may not result in a proportionally high protein content of white flour. Consequently, depending on the flour extraction rate, grain nitrogen measurements for crops that are exposed to high temperature during grain-filling may have a lower predictive value of flour functionality than for crops grown at lower temperatures. Since limiting nitrogen supply appeared to have the opposite effect, the increase in protein concentration gradient caused by high post-anthesis temperatures could be offset to a certain degree by decreasing the amount of nitrogen applied to the crop prior to anthesis. Although it is not possible to accurately predict weather conditions during grain-filling at the point of applying nitrogen to the crop during vegetative growth, data from this study may prove useful in cases where post-anthesis applications of nitrogen to wheat crops may have been scheduled and, more generally, in the context of years where higher summer temperatures are expected (for example, in coincidence with El Niño/North Atlantic Oscillation weather phenomena). Reduction of nitrogen input, however, may add to the reduction in yield already observed in wheat grown under higher temperatures.

Size distribution analysis of protein bodies in the grain further explained the nature of the observed gradient in protein concentration described in this experiment, and the distribution was characterised by a general trend of decreasing protein body size towards the central endosperm. However, variation in this general trend was observed, with the analysis of the earlier sampling timepoint showing an increase in mean protein body size towards the central endosperm after the initial decrease. Analysis of the histograms in Fig. 5 shows that this was due to an increase in the proportion of medium-sized protein bodies in the inner endosperm, accompanied by a decreased proportion of small protein bodies, with the frequencies of larger protein bodies showing minimal differences across the endosperm zones. It is possible that this effect was caused by the levels of starch in the grain, with lower levels of starch accumulated at the earlier sampling timepoint allowing the protein bodies to expand to a greater size in the central endosperm tissue.

The size distribution analysis revealed that protein bodies were generally larger at the later sampling timepoint, which is to be expected since grain protein production is ongoing during grain-filling, and smaller protein bodies will fuse to form larger protein bodies as they grow (Moore *et al.*, 2016). It was apparent that most of the measured protein bodies were very small, as shown in Fig. 5, with all treatments showing an abundance of small bodies and comparatively few larger bodies. However, it was in the frequency of large protein bodies that the differences were most apparent, with more larger protein bodies observed at the later sampling timepoint, and with distinct gradients in the proportions of the larger protein bodies across the endosperm zones.

## Conclusions

Both grain morphology and nitrogen concentration were affected by elevated post-anthesis temperature and restricted nitrogen supply. High post-anthesis temperature resulted in smaller grains with a higher concentration but same overall content of nitrogen in the mature grain, whilst limiting nitrogen supply resulted in both smaller and fewer grains being produced, and decreased both grain nitrogen concentration and nitrogen yield.

Furthermore, temperature and nitrogen supply interacted to affect both total protein distribution and protein body size distribution, as detected through image analysis of microscopy sections from grain at mid grain-filling. Elevated post-anthesis temperature affected both the gradient of total protein within the wheat grain endosperm, and the size distribution of protein bodies, with an increase in the steepness of the negative gradient observed between outer and inner endosperm tissue. Increasing nitrogen supply also had comparable, but less pronounced, effects on the gradient of both total protein and protein body size. Whilst we cannot comment on the changes to baking quality of individual mill streams that these gradients may produce, this study shows that high temperature during grain-filling has the capacity to reduce the protein yield of white flour, as proportionally more protein will be adhered to the aleurone layer, which is removed during the production of white flour. However, we also show that this potentially negative effect may be somewhat reduced by a reduction in the supply of nitrogen fertiliser to the plant prior to grain-filling.
